# Supplementary figures and images for: Dietary Interventions for Gout and Effect on Cardiovascular Risk Factors: A Systematic Review
Source: Nutrients. 2019 Dec 4;11(12):2955. doi: 10.3390/nu11122955 (PMC6950134; doi:10.3390/nu11122955)

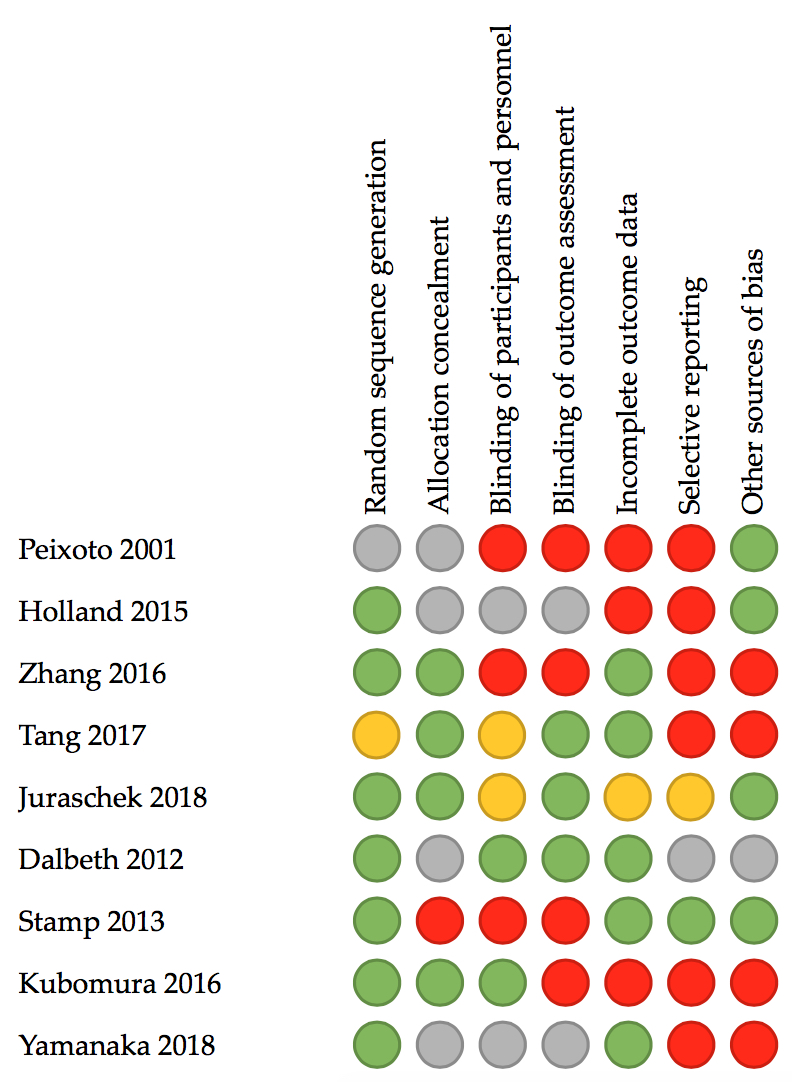

Supplement: Supplementary file 1 [file nutrients-11-02955-s001.zip › nutrients-629959-SI.jpg]
